# Associations between cervical disc degeneration and muscle strength in a cross-sectional population-based study

**DOI:** 10.1371/journal.pone.0210802

**Published:** 2019-01-25

**Authors:** Gentaro Kumagai, Kanichiro Wada, Hitoshi Kudo, Toru Asari, Daisuke Chiba, Seiya Ota, On Takeda, Kazushige Koyama, Shigeyuki Nakaji, Yasuyuki Ishibashi

**Affiliations:** 1 Department of Orthopaedic Surgery, Hirosaki University Graduate School of Medicine, Hirosaki, Aomori, Japan; 2 Department of Social Medicine, Hirosaki University Graduate School of Medicine, Hirosaki, Aomori, Japan; University of Alabama at Birmingham, UNITED STATES

## Abstract

The physical and biochemical factors related to cervical disc degeneration (CDD), which is involved in several spinal disorders, remain uncertain. We investigated associations between CDD and muscle strength in a general Japanese population. We used mid-sagittal-plane MRIs to assess CDD in 344 subjects recruited from participants in our community health-check project, and measured body mass index (BMI), skeletal muscle index (SMI), and muscle strength in the neck, trunk, hands, and legs. CDD was scored based on the prevalence and severity of intravertebral disc degeneration. Spearman correlation coefficients were used to evaluate whether the SMI or muscle-strength values were correlated with the disc degenerative score. Stepwise multiple linear regression analyses were then conducted with the CDD score as the dependent variable, and age, sex, BMI, and muscle strength as independent variables, for each gender. These analyses used the muscle-strength parameters that were found to be correlated with the CDD scores in the single correlation analyses. The CDD scores were similar in men and women. Men had significantly more muscle strength in the neck, trunk, hands, and legs. There was a significant negative corelation between the CDD score and the trunk strength in both sexes, handgrip in men, and leg strength in women in the single-variable correlation analysis. Including age and the limb- or trunk-muscle strength comprehensively, multiple linear regression analyses showed that age was the strongest factor that was independently associated with CDD in both sexes, and that the effects were attenuated by limb and trunk muscle strength.

## Introduction

Chronic neck pain and neck shoulder stiffness are common symptoms in the general population [1]. One study showed that radiographic degenerative changes in the cervical spine were associated with the severity of neck pain in a general population [2]. However, degenerative changes in the cervical spine are also common in asymptomatic individuals, challenging the simple concept of cause and effect [3]. The number of subluxations and the incidence and severity of degenerative changes in the cervical spine increase with age [4, 5]. Typical changes include osteoarthritis of the facets with reduced joint space and disc narrowing. Cervical myelopathy, a common degenerative spinal disease that interferes with normal activities [6], partly involves compression of the cervical spinal cord by spinal canal stenosis. Cervical spinal canal stenosis can arise from developmental canal stenosis, intervertebral disc protrusion into the spinal canal, or thickening of the ligamentum flavum [7]. Degenerative cervical changes can also appear in the intervertebral discs, and these changes progress with age [8] [9]. Epidemiologic studies have shown that the prevalence of cervical canal stenosis increases with age [10] [11]. Reported risk factors for cervical disc degeneration (CDD) include age [9] [2] [12], genetics [13], bone metabolism [12], smoking [14], metabolic syndrome [15], manual labor [16], and lumbar spinal disorders [17] [18].

Lumbar disc degeneration is associated with atrophy of the paraspinal muscles [19] [20]. In contrast, a study in Japanese asymptomatic subjects found no association between the cross-sectional area of cervical muscles and degeneration of the cervical discs [17]. Thus, the relationship between CDD and the strength of the neck, trunk, and extremities muscles in the general population remains unclear. Here, we hypothesized that muscle weakness is related to CDD. To test this hypothesis, we investigated the associations between CDD and the strength of various muscles in subjects recruited from the general population in a Japanese community. The obtained results may help clinicians determine specific preventive measures for disc degeneration.

## Methods

### Participants and study design

The subjects were recruited through a community-based public health project in a small city in northern Japan. For 10 years, this project has provided annual health checkups to the general population and made the services of physicians, surgeons, orthopedists, gynecologists, urologists, psychiatrists, dermatologists, and dentists available to the community. This group is suitable for a cohort study, because it is in a rural area with little change in the population. Each year, the program serves about 1000 city residents > 19 years of age [21]. Our research on neck complaints was conducted as part of this project.

In 2015, we recruited volunteers from the 1112 people who participated in the community health project, as described in detail previously [22]. All of the participants filled out questionnaires about their past medical history, lifestyle, fitness habits, occupational history, family history, and health-related quality of life. They also provided disease-specific information and described any symptoms related to the cervical spine and extremities. All the participants were examined for neurological status (deep tendon reflex), height (Ht), weight, individual body resistance, bone status including bone mineral density, and biochemical tests. Individuals were excluded if they had a history of stroke, cerebral bleeding, cervical spine trauma or surgery, or any systemic disease involving the cervical spine (such as rheumatoid arthritis), or if they did not complete the questionnaire. Cervical spine MRIs were evaluated randomly with respect to age. All volunteers gave informed, written consent before participating. This cross-sectional survey was approved by the ethics committee of the Hirosaki University Graduate School of Medicine.

### Measurement of muscle mass

Body resistance (R) was measured at 50 kHz using the Tanita MC-190 body composition analyzer (Tanita Co., Tokyo, Japan). Skeletal muscle mass (SM) was calculated using Janssen’s regression equation, which is based on the relationship between bioelectrical impedance analysis and SM measured by MRI [23], as follows: SM (kg) = [(Ht^2^/R × 0.401) + (gender × 3.825) + (age × −0.071)] + 5.102, where Ht is in centimeters, R in ohms, and age in years. Gender value is male = 1 and female = 0. The coefficient of determination (*r*^2^) in this regression equation was 0.86; the standard error of the estimate (SEE) was 2.7 kg or 9%. The skeletal muscle index (SMI) was calculated as SM/Ht^2^ × 10^2^ (kg/m^2^) to standardize differences influenced by height.

#### Assessment of muscle strength

To assess the associations between disc degeneration and muscle strength, trunk and leg strength were measured as described in detail previously [24] and neck and handgrip strength were additionally measured in this study. To measure the strength of the neck muscles, we used a device consisting of a torque machine with a MicroFET2 dynamometer (Nihon MEDIX Inc., Chiba, Japan). Isometric neck-muscle strength was measured with the subject in the prone position for extension and supine position for flexion as described in detail previously [25]. Isometric muscle strength was measured as peak torque (N) using the maximum pushing force on the pad of the MicroFET2. This torque value was adjusted by the subject’s body weight (N/kg).

Strength in the trunk muscles was measured using an iron frame combined with a QTM-06b as described in detail previously [24] [26]. Isometric trunk muscle strength was measured in both extension and flexion as peak torque (Nm) with the maximum pushing force on the QTM-06b. This torque value was adjusted by the subject’s body weight (Nm/kg).

Handgrip strength (in kg) was measured with a handheld dynamometer with the subject standing upright. The better of two trials was used for each hand.

Isometric muscle strength in the lower extremities was measured with an S-13129 (Takei Scientific Instruments Co., Ltd, Niigata, Japan) with the knee joint stabilized at a 90° angle as described in detail previously [24]. According to the determined arm length, 0.175 m, the peak force (kg) was used to calculate the peak torque (Nm/kg), adjusted by the subject’s body weight.

### MRI procedures

All MRI studies used a mobile MRI unit (Intera Achieva 1.5 T; Philips, Amsterdam, Netherlands) with a 1.5-Tesla (T) superconducting imager and phased array coils. Cervical spine MRIs were obtained with the subject in a supine position and the following imaging protocol: sagittal T2-weighted fast-spin echo (FSE): repetition time (TR) 4000 ms/echo, echo time (TE) 200 ms; field of view (FOV) 300 × 320 mm); and axial T2-weighted FSE: TR 4000 ms/echo; TE 120 ms; FOV 180 × 180 mm. Sagittal T2-weighted images were used to assess the intervertebral spaces from C2/3 to C7/T1.

#### Evaluation of CDD on cervical MRI

Intervertebral disc degeneration was evaluated on MRIs at all cervical levels from C3/4 to C7/T1, using Matsumoto’s classification system [8]. The signal intensity of the invertebral disc was graded as follows: Grade 0, as bright as or slightly less bright than the cerebrospinal fluid; Grade 1, markedly darker than the cerebrospinal fluid; Grade 2, no signal. Disc space narrowing was graded as a percentage of the height of a healthy upper disc, as follows: Grade 0, 100–75%; Grade 1, 75–50%; and Grade 2, less than 50%.

CDD severity was scored by adding the Matsumoto grades for signal intensity and disk space narrowing for all intervertebral sections from C2/3 to C7/T1. The total was defined as the degenerative score, with 0 corresponding to the normal cervical disc condition and 20 to the most severely degenerative disc condition [12].

### Statistical analysis

SPSS ver. 12.0J was used for data input and statistical calculations (SPSS Inc., Chicago, IL, USA). Differences in age, BMI, degenerative score, SMI, and muscle strength results between men and women were assessed by the Mann–Whitney *U* test, and differences in exercise habits and the prevalence of smoking were analyzed by the Chi-square test. Correlations between the degenerative score and age, SMI, or muscle strength were analyzed by Spearman’s rank correlation coefficient. Stepwise multiple linear regression analyses were conducted using the degenerative score as the dependent variable. The independent variables used were age, BMI, and a muscle-strength parameter, for each gender. For all analyses, a *P* value < .05 was considered significant.

## Results

Among the volunteers from our public health-project population, 151 men (mean age 54.2) and 193 women (mean age 55.5) were enrolled in the study. The youngest of the 344 participants was 20, and the eldest was 86 years old (Table 1). Table 1 summarizes the participants’ age, smoking, exercise habits, BMI, SMI, degenerative score, and muscle strength. There was no significant difference in age between the men and women. The men had a significantly higher prevalence of smoking (*P* < .0001) and a higher BMI (*P* < .001). Although there was no significant difference in mean degenerative score between the men and women, the men had a significantly higher mean SMI and had significantly more strength in the neck, trunk, hands, and legs (Table 1).

**Table 1:** Characteristics, degenerative score, and muscle strength of study participants.

			Men (*n* = 151)	Women (*n* = 193)	*P* value^b^
Age^a^, y			54.3 ± 15.1	55.6 ± 14.6	.432
		20–39	30	38	
		40–59	60	68	
		60–79	59	82	
		80–99	2	5	
Current Smoker, *n* (%)			33 (21.9)	18 (9.3)	< .0001^#^
Exercises regularly, *n* (%)			37 (24.5)	47 (24.4)	.916
BMI^a^, kg/m^2^			23.9 ± 3.3	22.3 ± 3.3	< .001*
SMI^a^, kg/m^2^			18.9 ± 1.6	15.4 ± 1.5	< .001*
Degenerative score^a^			8.4 ± 3.5	8.1 ± 3.4	.509
Neck strength^a^, N/kg			(*n* = 151)	(*n* = 191)	
	*Flexion*		1.2 ± 0.4	0.9 ± 0.5	< .0001*
	*Extension*		1.8 ± 0.6	1.6 ± 0.7	.003*
Trunk strength^a^, Nm/kg			(*n* = 125)	(*n* = 159)	
	*Flexion*		2.1 ± 0.7	1.6 ± 0.6	< .0001*
	*Extension*		5.2 ± 1.3	3.5 ± 1.2	< .0001*
Handgrip strength^a^, kg			(*n* = 126)	(*n* = 163)	
	*Right side*		40.5 ± 8.0	23.8 ± 4.7	< .0001*
	*Left side*		38.8 ± 7.9	23.0 ± 4.6	< .0001*
Leg strength^a^, Nm/kg			(*n* = 119)	(*n* = 151)	
	*Flexion*		1.5 ± 0.4	1.1 ± 0.3	< .0001*
	*Extension*		2.8 ± 0.8	2.0 ± 0.5	< .0001*

BMI, body mass index. SMI, skeletal muscle index.

^a^Mean ± S.D.

^b^Significant differences (*P* < .05) between values for men and women were calculated by *Mann-Whitney *U* or ^#^Chi-square test.

Single correlation analyses revealed a significant positive correlation between degenerative score and age for both sexes and SMI in women (Table 2) and significant negative correlations between the degenerative score and the strength of trunk flexion in both sexes, handgrip (both sides) in men, and handgrip (right side) and leg flexion and extension in women (Table 2).

**Table 2:** Relationships between degenerative score and muscle strength.

Partial correlation coefficient:		Degenerative score		
		*r*	*P* value	*n*
**Men**		* *	* *	
Age		0.637	< .0001*	151
SMI, kg/m^2^		−0.06	.935	151
Neck strength, kg				
	*Flexion*	0.102	.214	151
	*Extension*	-0.040	.629	151
Trunk strength, Nm/kg				
	*Flexion*	−0.247	.006*	125
	*Extension*	−0.011	.903	126
Handgrip strength, kg				
	*Right side*	−0.178	.046*	127
	*Left side*	−0.254	.004*	127
Leg strength, Nm/kg				
	*Flexion*	−0.177	.054	119
	*Extension*	−0.158	.087	119
**Women**				
Age		0.683	< .0001*	193
SMI, kg/m^2^		0.233	.001*	193
Neck strength, kg				
	*Flexion*	-0.048	.510	190
	*Extension*	-0.021	.776	190
Trunk strength, Nm/kg				
	*Flexion*	−0.272	.001*	158
	*Extension*	-0.051	0.523	157
Handgrip strength, kg				
	*Right side*	−0.169	.032	162
	*Left side*	−0.152	.054	162
Leg strength, Nm/kg				
	*Flexion*	−0.343	< .0001*	148
	*Extension*	−0.190	.020*	150

SMI: Skeletal muscle index. r = correlation coefficient. The relationship between degenerative score for Age, SMI and muscle strength were analyzed by Spearman’s rank partial correlation analysis. P-Values below 0.05* indicate significance.

Stepwise multiple linear analyses showed a significant correlation between the degenerative score and age in both men (B = 0.179, 95%CI: 0.139–0.218) and women (B = 0.162, 95%CI: 0.128–0.196), but not between the degenerative score and any muscle strength measure (Table 3). In this analysis, the muscle-strength parameters were those found to be associated with CDD in the single correlation analysis (trunk flexion and handgrip in men, and trunk flexion and leg strength in women).

**Table 3:** Stepwise multiple regression analysis relative to degenerative score^a^.

Logistic regression (degenerative score)		B	β	95% CI	*P* value
**Male**					
	Age	0.179	0.802	0.139 to 0.218	< .0001
	BMI, kg/m^2^	0.106	0.107	-0.065 to 0.276	0.222
	Trunk strength, Nm/kg				
	*Flexion*	0.460	0.098	-0.334 to 1.255	0.253
	Handgrip strength, kg				
	*Right side*	0.047	0.113	-0.054 to 0.147	0.360
	*Left side*	-0.082	-0.192	-0.183 to 0.020	0.114
**Female**					
	Age	0.162	0.704	0.128 to 0.196	< .0001
	BMI, kg/m^2^	0.095	0.089	-0.066 to 0.256	0.247
	Trunk strength, Nm/kg				
	*Flexion*	0.012	0.002	-0.763 to 0.786	0.977
	Leg strength, Nm/kg				
	*Flexion*	-0.033	-0.003	-2.092 to 2.026	0.975
	*Extension*	0.292	0.048	-0.762 to 1.345	0.585

B, regression coefficient; β, standardized regression coefficient; *r*^2^, coefficient of determination (adjusted).

^a^Stepwise multiple regression analysis was performed by gender using the degenerative score as the dependent variable and age, BMI, and muscle strength as independent variables. The muscle-strength parameters were those found to be correlated with the degenerative score in the single correlation analysis (trunk flexion and handgrip in men, and trunk flexion and leg strength in women).

## Discussion

To our knowledge, this is the first community-based survey of associations between CDD and muscle strength in a rural Japanese population. We showed that the strength of trunk flexion in men and women, hand grip in men, and leg strength in women were negatively correlated with CDD scores in single-variable correlation analyses. Multiple linear regression analyses that comprehensively included age and limb or trunk muscle strength showed that age was the strongest independent factor associated with CDD in both sexes, while the associations of limb and trunk muscle strength were attenuated.

Our findings that age was the strongest determinant of CDD in both sexes is in agreement with an MRI based study in the Japanese population.They found that progression of cervical-spine degeneration. Another study using MRI showed that the progression of cervical-spine degeneration on MRI was frequently observed during a 10-year period, along with symptom development in healthy volunteers [9]. In that study, the only factor related to the progression of cervical-spine degeneration was age. In addition, a genetic association study found age to be the most significant determinant of lumbar disc degeneration [27]. We previously assessed CDD on X-rays and demonstrated that age was correlated with CDD [2]; we also found signficant correlations between CDD and cross-linked N-telopeptide of type 1 collagen as a bone metabolism marker and isoleucine as an amino acid marker in men, and lysine as an amino acid marker in a general community population [12]. These studies collectively indicate that in assessing the cervical spine, clinicians should pay particular attention to age-related bone metabolism factors that may be associated with disc degeneration.

In the present study, single-variate correlation analyses showed that the muscle strength of trunk flexion in both sexes, hand grip in men, and leg strength in women were negatively correlated with CDD. Okada et al. reported that CDD is associated with changes in the cross-sectional area of the extensor muscles of the cervical spine on MRIs [17], but not with changes in the extensor muscle volume. Hakkaku et al. reported that the repeated crash forces experienced by athletes subjected to contact and external stress are risk factors for CDD [25]. Other studies have investigated associations between disc degeneration and muscles in the lumbar spine [13] [28]. Videman et al. reported that lumbar disc degeneration was associated more strongly with body weight, lifting strength, and axial disc area than with a history of physically demanding work or activities [28]. Therefore, physical loading and mechanical stress may be crucial steps in the progression of discdegeneration. We recently reported that lumbar spondylosis and the muscle strength of the trunk are associated with locomotive syndrome, in which individuals exhibit deteriorating locomotorium and thus may require nursing or other support [24]. Taken together, weaker trunk muscle strength has been considered to be related to the progression of CDD. Notably, in the present cross-sectional study when we considered the association among CDD, age, and muscle strength at the same time, we confirmed that the muscle strength of the limb and trunk decreased linearly with age, and that the correlation between CDD and muscle strength was attenuated in the middle to elderly population. Based on the current results, a longitudinal study of the cervical spine following the same individuals might provide clues to the underlying cause of the disc degeneration, as long as researchers are careful to be aware of the strong linearity between age and muscle strength.

This study had several limitations that should be noted. First, we did not evaluate the duration, region, or distribution of neck symptoms. Progressive structural changes over a long period of time are likely to correlate significantly with future clinical symptoms. Second, our study population was geographically limited to a district with many farming villages, and thus may not be representative of Japan as a whole. Therefore, lifestyle factors such as occupation and hobbies should be considered, and caution should be applied when generalizing our results to other populations. Third, we did not investigate the subjects’ level of education or other aspects of their medical history, such as bone metabolism [12], metabolic syndrome [15], or lumbar spinal disorders [17] [18] in this study.

In the present study, the strength of trunk flexion in men and women, hand grip in men, and leg strength in women were negatively correlated with CDD scores in a single-variate correlation analysis. On the other hand, multiple linear regression analyses in which age and muscle strength were included simultaneously, showed that age was the most significant determinant of CDD, and that the effects of muscle strength were attenuated and not significant. Although a longitudinal study is needed to determine the strategies by which enhancing muscle strength will prevent CDD, our current findings suggest that researchers should take the colinearity of age and muscle strength into account when they examine these strategies epidemiologically.
